# Regional and Developmental Differences in Na^+^ Currents in Vestibular Primary Afferent Neurons

**DOI:** 10.3389/fncel.2018.00423

**Published:** 2018-11-14

**Authors:** Frances L. Meredith, Katherine J. Rennie

**Affiliations:** ^1^Department of Otolaryngology, University of Colorado School of Medicine, Aurora, CO, United States; ^2^Department of Physiology & Biophysics, University of Colorado School of Medicine, Aurora, CO, United States

**Keywords:** calyx, semicircular canal, crista, 4,9-anhydrotetrodotoxin, tetrodotoxin, Na^+^ channel

## Abstract

The vestibular system relays information about head position *via* afferent nerve fibers to the brain in the form of action potentials. Voltage-gated Na^+^ channels in vestibular afferents drive the initiation and propagation of action potentials, but their expression during postnatal development and their contributions to firing in diverse mature afferent populations are unknown. Electrophysiological techniques were used to determine Na^+^ channel subunit types in vestibular calyx-bearing afferents at different stages of postnatal development. We used whole cell patch clamp recordings in thin slices of gerbil crista neuroepithelium to investigate Na^+^ channels and firing patterns in central zone (CZ) and peripheral zone (PZ) afferents. PZ afferents are exclusively dimorphic, innervating type I and type II hair cells, whereas CZ afferents can form dimorphs or calyx-only terminals which innervate type I hair cells alone. All afferents expressed tetrodotoxin (TTX)-sensitive Na^+^ currents, but TTX-sensitivity varied with age. During the fourth postnatal week, 200–300 nM TTX completely blocked sodium currents in PZ and CZ calyces. By contrast, in immature calyces [postnatal day (P) 5–11], a small component of peak sodium current remained in 200 nM TTX. Application of 1 μM TTX, or Jingzhaotoxin-III plus 200 nM TTX, abolished sodium current in immature calyces, suggesting the transient expression of voltage-gated sodium channel 1.5 (Nav1.5) during development. A similar TTX-insensitive current was found in early postnatal crista hair cells (P5–9) and constituted approximately one third of the total sodium current. The Nav1.6 channel blocker, 4,9-anhydrotetrodotoxin, reduced a component of sodium current in immature and mature calyces. At 100 nM 4,9-anhydrotetrodotoxin, peak sodium current was reduced on average by 20% in P5–14 calyces, by 37% in mature dimorphic PZ calyces, but by less than 15% in mature CZ calyx-only terminals. In mature PZ calyces, action potentials became shorter and broader in the presence of 4,9-anhydrotetrodotoxin implicating a role for Nav1.6 channels in firing in dimorphic afferents.

## Introduction

The vestibular system of the inner ear detects and signals information about head position and acceleration. In response to mechanical stimuli at the hair bundle, vestibular hair cells release transmitter onto afferent dendrites and resulting postsynaptic changes sculpt action potential firing in afferent neurons. Voltage-gated Na^+^ and K^+^ channels are necessary for action potential generation and propagation, but ion channel sub-types and their roles within specific groups of vestibular afferents are not resolved. We therefore explored the identities and roles of Na^+^ channel subunits underlying Na^+^ currents (*I*_Na_) in calyx-bearing vestibular afferent terminals in developing and mature gerbil crista. We hypothesized that regional differences in Na^+^ channel subunit expression across the crista could contribute to the known heterogeneity of firing patterns observed in mature vestibular afferents (Goldberg, 2000).

Vestibular afferents are bipolar neurons with cell bodies in the vestibular ganglion. Processes from one pole of the cell body form specialized terminals contacting presynaptic mechanosensitive hair cells within the neuroepithelium. Extensions from the opposite pole terminate within the brainstem and cerebellum. The vestibular nerve in the gerbil contains ∼4000 afferent fibers (Kevetter and Leonard, 2002) which carry information from hair cells in the five vestibular sensory end organs of each ear to the brain. Afferents within different zones of vestibular epithelia have distinct morphological and firing properties. In mammalian species, calyx-only afferents contact one or more type I hair cells in central locations of the neuroepithelia, whereas bouton afferents contact only type II hair cells in peripheral regions. Dimorphic afferents supply type I and type II hair cells through calyx and bouton terminals, respectively, and are found in both regions. In both crista and otolith organs, centrally located afferents exhibit spontaneous action potentials with highly variable spike intervals, whereas in peripheral zones (PZs), afferent firing is highly regular (Goldberg, 2000; Eatock and Songer, 2011). Thin transverse slices of the crista allowed us to investigate Na^+^ channels in central zone (CZ) and peripheral zone (PZ) calyx-bearing afferents. In mature cristae, some afferents were labeled and identified as calyx-only fibers, restricted to CZ and contacting exclusively type I hair cells, or dimorphic fibers found in both zones and contacting both type I and type II hair cells.

Na^+^ channel alpha (α) subunits (Nav1.1–1.9) have variable sensitivities to block by the marine bacterial toxin tetrodotoxin (TTX). Some Nav-mediated currents are blocked by nanomolar concentrations, whereas others are not blocked by even high concentrations of TTX (Renganathan et al., 2002; Black et al., 2008). Voltage-gated Na^+^ channels that are blocked by nanomolar concentrations of TTX are classified as TTX-sensitive (Nav1.1, Nav1.2, Nav1.3, Nav1.4, Nav1.6, and Nav1.7 channels). Nav1.8 and 1.9 channels are resistant to block by even micromolar concentrations of TTX (Sivilotti et al., 1997; Cummins et al., 1999) and are classified as TTX-resistant, whereas Nav1.5 channels have IC50 values for TTX block in the micromolar range (Goldin, 2001) and can be classified as TTX insensitive.

Immunohistochemical probing for Na^+^ channels in vestibular epithelia has demonstrated unforeseen patterns of Na^+^ channel distributions within calyx afferent endings, as well as their expected distribution along the axons of vestibular primary afferent fibers. Nav1.5-like staining was associated with hair cells, with the inner face of calyx terminals and in afferent fibers below the epithelium in rat vestibular organs (Wooltorton et al., 2007; Lysakowski et al., 2011). Nav1.6 staining was seen at the heminode and nodes of dimorphs. Pan-Nav antibodies, targeting an epitope common to all Nav1 α subunits, showed staining patterns on heminodes and on both faces of all calyces (Lysakowski et al., 2011). A variety of Nav subtypes was also recently reported in the rat vestibular ganglion (Liu et al., 2016).

Action potentials are generally thought to originate at the axon initial segment, a key site in neurons where several ion channels are clustered and before terminals are wrapped in myelin (Bender and Trussell, 2012). However, calyx-bearing afferents have an unusual morphology and how and where spikes are initiated within these terminals is unclear. Here, we used whole cell recordings to determine which Nav1 isoforms may allow the formation and propagation of action potentials in primary vestibular neurons. We investigated the electrophysiological expression and identity of Nav α subunits in vestibular afferent terminals based on their biophysical properties and sensitivity to pharmacological blockers. Our goals were to determine the relative contributions of TTX-sensitive and non-TTX-sensitive Na^+^ channels to macroscopic currents and to action potential firing in fully formed vestibular afferents, and to investigate what changes, if any, occur during maturation.

## Materials and Methods

### Ethics Statement

Animal procedures were approved by the University of Colorado’s Institutional Animal Care and Use Committee and followed National Institutes of Health guidelines. Mongolian gerbils (*Meriones unguiculatus*, male and female) were bred in an in-house colony and used in experiments between postnatal day (P)5 and P31. Gerbils were deeply anesthetized with an intraperitoneal injection of ketamine (200 mg kg^−1^) and xylazine (20 mg kg^−1^) mixed in normal saline, decapitated, and the brain removed to expose the vestibular labyrinth. The three ampullae, containing cristae, were then carefully removed from the bony canals from each ear.

### Crista Slices

**FIGURE 1:**
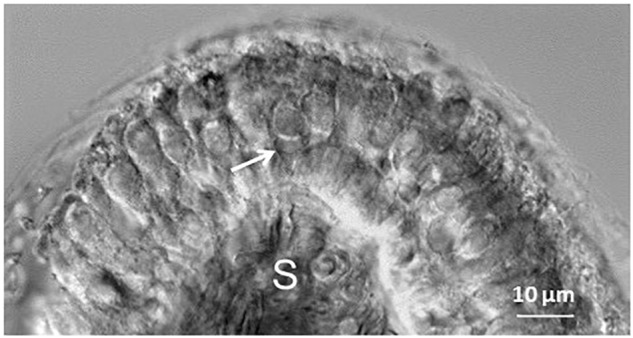
Differential interference contrast image of a transverse slice cut through the mid region of a P11 gerbil crista. Hair cells within the neuroepithelium are visible above the stroma (S). Arrow indicates a double calyx terminal contacting two type I hair cells in the central zone of the crista. Myelinated axons are visible within the stroma.

Ampullae were maintained in Leibovitz’s L-15 medium, pH 7.4–7.45, osmolality 300–305 mOsm (kg distilled water)^−1^ with 0.5 mg ml^−1^ bovine serum albumin (BSA) for a minimum of 50 min at room temperature (21–24°C). Each ampulla was trimmed with iris scissors and placed in 4% low gelling temperature agarose (2-hydroxyethylagarose, Type VII, Sigma-Aldrich, St Louis, MO, United States) in Dulbecco’s phosphate-buffered saline (in mM): KCl (2.7), KH_2_PO_4_ (1.5), NaCl (137.9), and Na_2_HPO_4_ (8.1). A block containing the crista was glued to the stage of a tissue slicer (Vibratome 3000 EP or Leica VT1200S) and placed in L-15 solution. Crista sections were cut transversely at 100–110 μm. Individual slices were placed in a recording chamber filled with external solution. L-15 was the standard external solution, but in some experiments, a “0 K^+^” HEPES solution (in mM): NaCl (150), MgCl_2_ (1.8), CaCl_2_ (1.3), HEPES (10), and glucose (10) was used and in others a “low Na^+^/0 K^+^” HEPES solution was used (in mM): NaCl (80), CsCl (5.4), MgCl_2_ (2.5), tetraethylammonium chloride (TEACl) (70), CaCl_2_ (1.3), glucose (10), and HEPES (5). Slices were viewed on an Olympus upright microscope (BX51WI) with water immersion objectives (40× and 60×) and differential interference contrast optics (Figure 1). In a subset of experiments, 50 μM Alexa Fluor 488 hydrazide, Na^+^ salt (Molecular Probes, Thermo Fisher Scientific, Waltham, MA, United States) was included in the patch electrode solution to label nerve terminals and distinguish dimorphic terminals contacting type I and type II hair cells, from calyx-only terminals contacting solely type I hair cells. Following electrophysiological recordings, the electrode was withdrawn from the intact calyx and fluorescent images captured to visualize the filled terminal field (Figure 3). Differential interference contrast and fluorescent images were obtained with a digital camera (Retiga R3, QImaging, Surrey, BC, Canada) and Ocular software (QImaging, Surrey, BC, Canada).

### Cut Peripheral End

The neuroepithelium widens at each end of the crista and the CZ ends just before the widest point (Desai et al., 2005). The region beyond the widest point, the planum, therefore contains only PZ calyces. In some cases, micro-dissection scissors were used to cut each planum region from the rest of the crista, forming two “cut ends” (Meredith and Rennie, 2015). The planum was anchored to the bottom of the recording dish with a fine minutien pin and recordings made from PZ calyces.

### Immature Cells

To record from immature hair cells (P5–9), cristae were incubated in L-15/BSA and then placed in a large drop of L-15 in the center of the recording chamber. A fine probe was used to mechanically dissociate the crista as previously described (Rennie and Streeter, 2006). Isolated hair cells were allowed to settle onto the bottom of the chamber (10 min) and the chamber was then topped up with L-15 to a volume of ∼1.5 ml. Isolated cells were viewed under an Olympus upright microscope (BX50WI or BX51WI) and electrophysiological recordings were made from type I hair cells identified by the presence of IK_LV_, the low voltage-activated K^+^ current typical of type I hair cells. The dissociated preparation also yielded a small number of calyx terminals still attached to their type I hair cell, and these were used in a minority of recordings from immature calyces. The majority of recordings from immature calyces were made from slices, but no distinction was made between CZ and PZ calyces since electrophysiological properties are not yet mature. Calyx afferents begin to form cup-shaped terminals around type I hair cells during the first postnatal week in mice (Rüsch et al., 1998). In mature gerbil, calretinin is a selective label of calyx-only afferents in the apical (central) region of the crista ampullaris (Leonard and Kevetter, 2002; Desai et al., 2005). In P4 mice, only a few fibers were reported to be calretinin positive, whereas by the third postnatal week, calretinin immunostaining in calyces was similar to adults (Dechesne et al., 1994). In addition, electrophysiological properties of hair cells and their afferents undergo maturation during the first postnatal weeks. We therefore refer to hair cells and calyces at ages P5–14 as “immature.” In a group of older animals, calyx recordings were made at ages ≥P20 and deemed “mature.”

### Electrophysiological Recordings

We recorded from as many neurons as possible from slices, cut ends and the dissociated preparation to minimize the number of animals used. Patch pipettes were pulled from capillary glass tubing (G85150T-3; Warner Instruments, Hamden, CT, United States) on a micropipette puller (P-97, Sutter Instruments, San Rafael, CA, United States), heat polished (Narishige M830 microforge (Narishige International USA, East Meadow, NY, United States) and coated near their tips with Silicone elastomer (Sylgard 184, Dow Corning, Midland, MI, United States) to reduce capacitance. Electrode solution for recording sodium currents (*I*_Na_) contained (in mM): CsF (120), CsCl (10), MgCl_2_ (2), NaCl (2), HEPES (10), glucose (3), and ethylene glycol tetraacetic acid (EGTA) (10) pH 7.4 adjusted with CsOH, osmolality 300–305 mOsm (kg distilled water)^−1^, adjusted with mannitol. For recording action potentials in current clamp, CsF and CsCl in the electrode solution was replaced with equimolar KF and KCl and pH adjusted with KOH. Electrode open tip resistance was 2–6 MΩ. Gigaseals were formed on the outer face of calyces and whole cell recordings made at room temperature (21–24°C) using a patch amplifier (Axopatch-1D or 200B, Molecular Devices, Sunnyvale, CA, United States) connected to a PC through an A/D converter (Digidata 1320A or 1440A, Molecular Devices). Liquid junction potentials were calculated using Clampex 10.3 (Molecular Devices) and subtracted off-line. Following membrane breakthrough, we consistently observed an increase in peak *I*_Na_ or “run up” during the first few minutes of recording in voltage clamp. Therefore, in most experiments, cells were perfused continuously with external solution using a Gilson (Gilson, Inc. Middleton, WI, United States) Minipuls 3 peristaltic pump for a minimum of 5 min at a rate of 0.5–1 ml min^−1^ to obtain steady baseline control values before perfusion of drugs onto slices or isolated cells. In a few experiments, drugs were applied by rapid replacement of bath solution using a transfer pipette. For experiments in current clamp, brief hyperpolarizing steps (−25 to −80 pA, duration 25–100 ms) were used to evoke action potentials.

### Drugs

Stock solutions of Jingzhaotoxin-III (JZTX-III) and TTX (Alomone Labs, Jerusalem, Israel) dissolved in deionized water were stored at −20°C until the day of use. Stock solutions of 4,9-anhydrotetrodotoxin (4,9-ah-TTX, Alomone Labs) were prepared in ethanol, stored at −20°C and used within 3 weeks.

### Data Analysis/Statistics

Electrophysiological data were analyzed using pClamp 10 (Molecular Devices, **RRID**SCR_23) and Sigmaplot 11 (Systat Software, San Jose, CA, United States, **RRID**SCR_003210). The voltage-dependent inactivation plot (Figure 4B) was fit with a Boltzmann function of the form:

(1) $$I/I_{\max}=1/1+\exp\left[\frac{V-V_{1/2}}{S}\right]$$

where *V* is the conditioning potential, *V*_1/2_ is the half-maximum inactivation potential, and *S* determines the slope factor for inactivation.

Evoked action potential data were analyzed using MiniAnalysis software (v 6.0.3, Synaptosoft, Decatur, GA, United States, **RRID**SCR_002184), and action potentials were aligned by rise time. Statistical significance was determined using the Students’ *t*-test (different populations) and paired *t*-test (same population; before and after). Mean values are given followed by standard deviation (SD). A result was considered significant when *P* < 0.05. In the figures, *P* values between 0.05 and 0.01 are summarized above dot plots with one asterisk, those less than 0.01 with two asterisks and those less than 0.001 with three asterisks. Exact *P* values are given in figure legends or text.

## Results

### General

Cristae were sliced in transverse sections at 100–110 μM in preparation for whole cell patch clamp recordings (Figure 1). The central third of the saddle-shaped crista slice corresponds to the CZ and adjacent slopes are designated as PZ (Desai et al., 2005; Meredith and Rennie, 2015) (see Desai et al., 2005, Figure 2, for a schematic diagram). Previously we reported slice recordings from gerbils aged P17–P33 (Meredith and Rennie, 2015), but here we obtained additional data from cristae from a group of animals at younger ages (P5–14). Figure 1 shows an example of a calyx contacting two type I hair cells in the CZ of a P11 crista (arrow).

**FIGURE 2:**
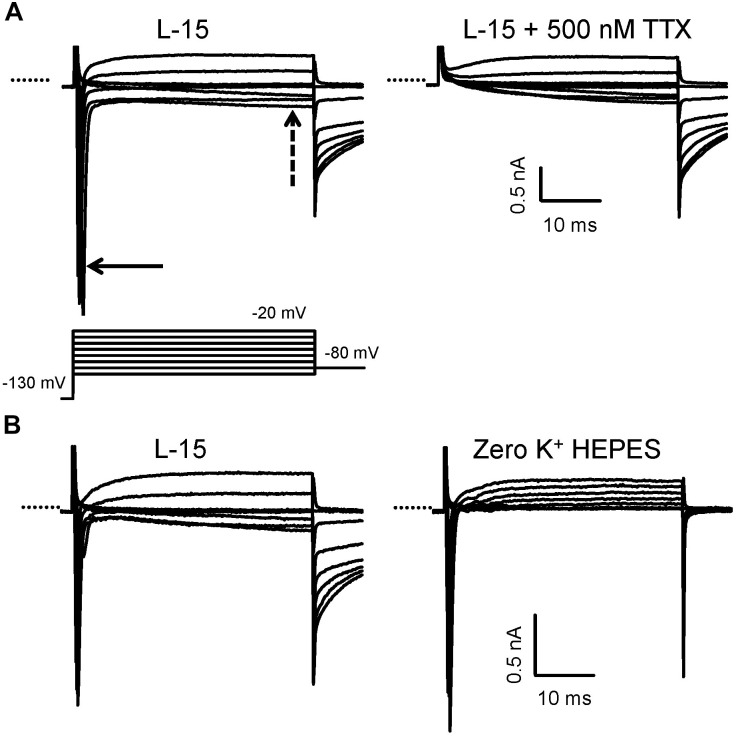
Isolation of Na^+^ currents in whole cell voltage clamp in calyces from the mature crista. **(A)** A family of currents was evoked in response to the standard voltage protocol consisting of a 40 ms step to –130 mV from a holding potential of –80 mV, followed by a series of 40 ms depolarizing steps in 10 mV increments (last part of protocol shown). Large and rapid inward currents (solid arrow) developed at steps to membrane potentials above –70 mV and were followed by a slowly developing inward current (dashed arrow) in standard L-15 solution. At more positive potentials, the inward current became outward. Perfusion with 500 nM TTX (right) abolished the transient inward current, indicating it was carried by TTX-sensitive Na^+^ channels, but the sustained inward current remained. PZ calyx, P25. **(B)** Control currents in L-15 in another cell followed by perfusion with zero K^+^ HEPES solution revealed that *I*_Na_ persisted, but the slow inward current was abolished. CZ calyx, P22. Electrode solution contained Cs^+^. In this and subsequent figures, capacitance transients have been truncated for clarity and the dotted line indicates zero current level.

Inward Na^+^ currents were recorded from PZ and CZ calyx terminals in slices and PZ terminals in cut ends. Some recordings from immature cristae were also obtained from type I hair cells or calyx terminals dissociated along with their type I hair cells. Patch electrodes targeted the unmyelinated basal surface of the calyx outer face for formation of gigaseals. Extracellular and intracellular solutions were designed to minimize inward and outward currents carried by K^+^ and to isolate Na^+^ currents. Using Cs^+^-based electrode solutions, all mature calyces from both zones showed rapid and transient inward currents at membrane potentials more positive than −70 mV (Figure 2). We have previously shown that similar currents in solitary calyces are abolished by removal of external Na^+^ and are blocked to a large extent by 100 nM TTX (Rennie and Streeter, 2006; Dhawan et al., 2010). In addition to the transient current, a small sustained inward current was present above −60 mV that was not blocked by 500 nM TTX (Figure 2A, right). Perfusion with 0 K^+^ HEPES solution removed the slow inward current indicating it was carried by K^+^ (Figure 2B). Subsequently, several experiments were performed in 0 K^+^ HEPES in order to better isolate *I*_Na_. In all these experiments, the slow inward current was removed, but a small residual outward current remained in 0 K^+^ HEPES solution which may be carried by Cs^+^. To further isolate *I*_Na_, some experiments were performed in a lowered Na^+^ extracellular solution, similar to that used to record *I*_Na_ in vestibular ganglion cells (Liu et al., 2016), where 70 mM of Na^+^ was replaced by TEACl.

### Regional Differences in *I*_Na_ in Calyces of the Crista

**FIGURE 3:**
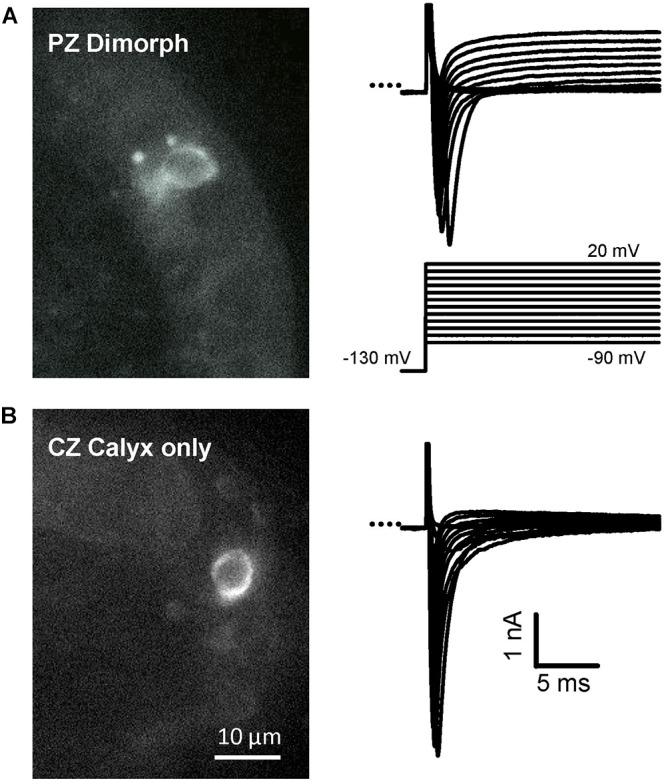
Examples of recordings from identified terminals in different zones. Alexa Fluor 488 was included in the electrode solution to label afferent endings in crista slices. **(A)** Dimorphic PZ afferent (left image, P27) with calyx and bouton terminal endings seen as a bright circle and two puncta with associated Na^+^ currents in response to the voltage protocol. **(B)** Calyx-only terminal in the central crista region (left image, P25). Corresponding Na^+^ currents recorded in voltage clamp are shown on the right. *I*_Na_ was isolated using internal Cs^+^ and 0 K^+^ HEPES solution. A small outward current persisted in the PZ cell but was minimal in the CZ cell. Scale bar shown in **(B)** apply to both **(A,B)**.

**FIGURE 4:**
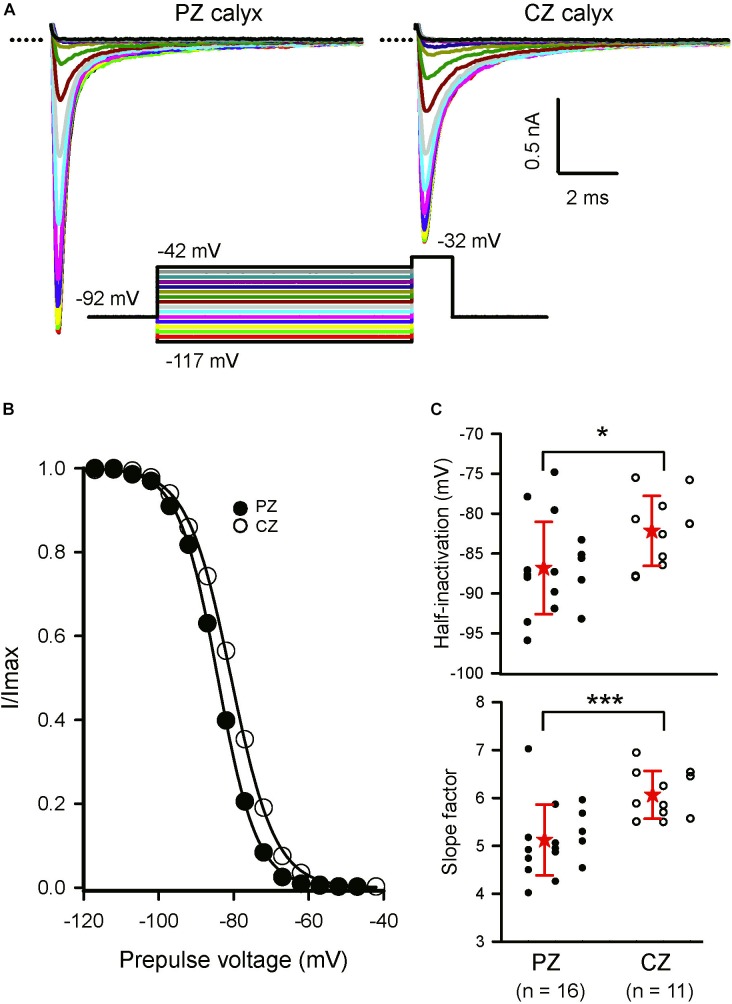
Inactivation of Na^+^ currents in regionally identified calyces. **(A)** Inactivation of *I*_Na_ in a PZ (P22) and CZ calyx-only afferent (P25). Currents shown are at a test step to –32 mV. Entire voltage protocol (shown below) consisted of prepulses from a holding potential of –92 mV to potentials between –117 and –42 mV in 5 mV increments before the test step. The largest currents were evoked following the most negative prepulses. **(B)** Currents were normalized to maximum inward current, plotted as a function of prepulse voltage and fit with a Boltzmann curve (Eq. 1). *V*_1/2_ inactivation was –84.2 mV in the PZ cell and –80.7 mV in the CZ cell. Slope factor was smaller in the in the PZ calyx (5.2) compared to the CZ calyx (5.9). **(C)** Mean *V*_1/2_ inactivation and slope values are compared for calyces from the two different zones. Dots represent individual calyx responses, stars the mean, and error bars the standard deviation of the mean. Mean half-inactivation in PZ calyces was –86.8 (5.8) mV (*n* = 16), significantly more hyperpolarized than in CZ calyces [–82.2 (4.4); *n* = 11; *P* = 0.024, *t-test*]. Mean slope factor in PZ calyces was 5.1 (0.7) (*n* = 16), significantly less than the value of 6 (0.5) in CZ calyces (*n* = 11; *P* < 0.001, *t-test*). ^∗^*P* < 0.05, ^∗∗∗^*P* < 0.001. Numbers below plots indicate number of cells for each group. External solution was 0 K^+^ and contained either 80 mM Na^+^ or 150 nM Na^+^. Electrode solution was Cs^+^-based.

Calyces form part of dimorphic arbors in PZ, but can exist as unmixed “calyx-only” or dimorphic terminals in CZ. To distinguish calyx-only afferents from dimorphic afferents, the fluorescent dye Alexa 488 was included in the patch electrode solution and terminals visualized in crista slices following electrophysiological recordings. Figure 3 shows Alexa-labeled calyx terminals and associated *I*_Na_ recorded from a mature (P27) PZ dimorph terminal (Figure 3A) with calyx and boutons and a mature (P25) CZ calyx-only ending (Figure 3B). The external solution was 0 K^+^ HEPES. Both cells showed rapidly activating and rapidly inactivating inward currents in response to the standard voltage protocol. Although Cs^+^ was present in the patch electrode solution, a small outward current persisted in the PZ calyx which was typical of mature PZ cells. By comparison, little outward current was present in CZ calyx-only cells. This suggests Cs^+^-permeability of a subset of K^+^ channels and is likely due to differences in K^+^ channel expression between zones (Meredith and Rennie, 2015; Meredith et al., 2015). Voltage-dependent inactivation of Na^+^ channels is removed at hyperpolarized membrane potentials and a different voltage protocol was used to determine inactivation characteristics of *I*_Na_ in both zones as shown in Figure 4. A comparison between zones revealed that the mean V_1/2_ inactivation for *I*_Na_ was significantly more negative in PZ calyces compared to CZ calyx-bearing afferents and the slope factor was greater for CZ calyx-bearing afferents (Figures 4B,C). In addition, *I*_Na_ showed significantly slower inactivation kinetics in identified CZ calyx-only afferents (identified by fluorescent fill). The decay of *I*_Na_ following the peak was well fitted with a double exponential function of the form:

(2) $$I(t)=\alpha_{1}\exp(-t/\tau_{1})+\alpha_{2}\exp(-t/\tau_{2})$$

where τ_1_ and τ_2_ are the fast and slow time constants.

In response to a voltage step from −130 to −35 mV, weighted τ was slower in P20–27 calyx-only afferents [1.2 (0.31) ms; *n* = 14] compared to P20–31 PZ dimorphic afferent calyx endings [0.95 (0.35) ms; *n* = 16; *P* = 0.04, *t-test*, data not shown].

Lastly, in identified mature (P20–27) calyces, all bathed in 0 K^+^ HEPES, mean *I*_Na_ amplitude (−50 mV step) was −4.1 nA (1.4), in PZ calyces (*n* = 8), significantly greater than in CZ calyx-only afferents (−2.62 (0.97) nA, *n* = 9, *P* = 0.008, data not shown). These biophysical observations of *I*_Na_ suggested that different Na^+^ channel subunits might contribute to *I*_Na_ in the different crista locations and this was probed further with Na^+^ channel blockers.

### TTX-Sensitive Currents in Mature Calyces

**FIGURE 5:**
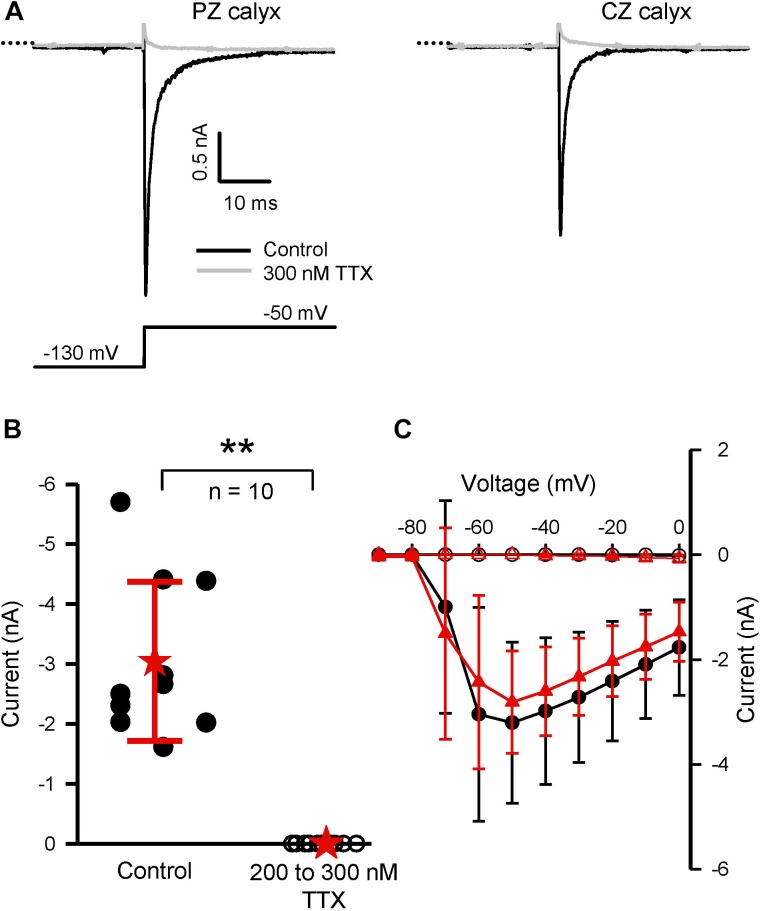
TTX is an effective blocker of *I*_Na_ in mature PZ and CZ calyces in the fourth postnatal week. **(A)** TTX block (300 nM) of current in a PZ and CZ cell (both aged P22) following a prepulse to –130 mV and voltage step to –50 mV. **(B)** Plot summarizing the effect of TTX on 10 calyces (five PZ and five CZ cells) aged P21–P25. At 200 or 300 nM (data pooled from 10 cells), TTX completely abolished the inward current measured at –50 mV. ^∗∗^*P* < 0.01. **(C)** IV plot shows that TTX completely blocked *I*_Na_ in the five CZ cells (red triangles; filled for control, open for block in TTX) and the five PZ cells (black circles; filled for control, open for block in TTX). External solution was L-15 and electrode solution Cs^+^.

Many, but not all, of the known Na^+^ channel alpha subunits are highly sensitive to block by TTX. We tested for the presence of TTX-sensitive currents using a range of concentrations of extracellular TTX (100–1,000 nM TTX). TTX-resistant Na^+^ currents are reported to persist in concentrations of TTX as high as 1 μM TTX, whereas moderately TTX-sensitive Na^+^ currents should be abolished by 1 μM, but not by nM TTX concentrations (Goldin, 2001). As shown in Figure 5, TTX concentrations between 200 and 300 nM were effective at completely blocking *I*_Na_ in both PZ and CZ calyces at P21–25. At these concentrations, TTX should block all TTX-sensitive Na^+^ channel subunits and the absence of any residual current suggests that Nav1.5 channels and TTX-resistant (Nav1.8 and 1.9) channels are absent. Lower concentrations (50 and 100 nM TTX) were also effective at blocking *I*_Na_ by >95% (*n* = 8, P17–29, data not shown).

### TTX-Insensitive Currents in Early Postnatal Calyces

**FIGURE 6:**
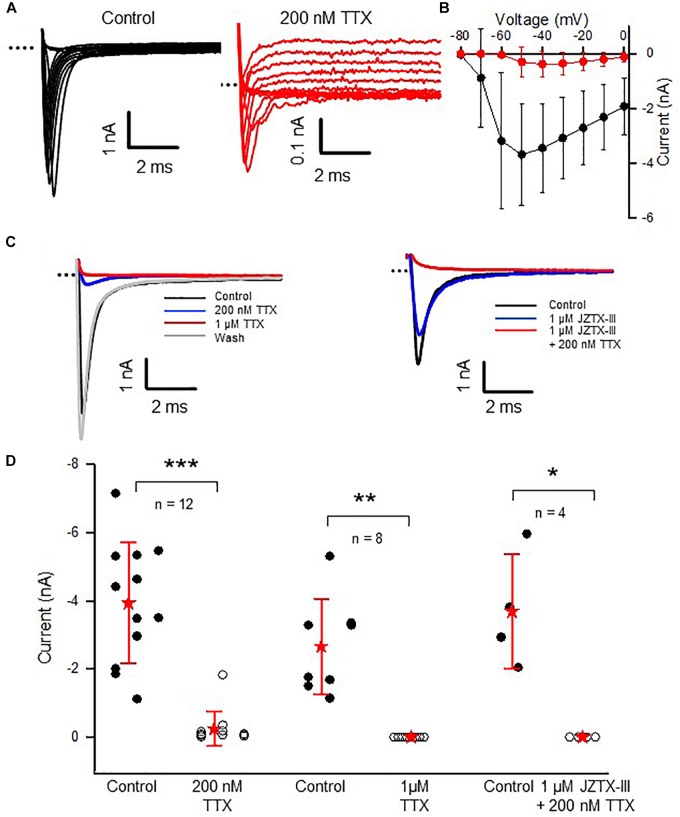
A TTX-insensitive current is present in immature calyces. **(A)** Control *I*_Na_ and currents remaining in the presence of 200 nM TTX at membrane potentials above –60 mV in a P8 calyx (note different current scales for left and right panels). Voltage protocol similar to Figure 2: a 40 ms step to –130 mV from a holding potential of –80 mV was followed by a series of 40 ms depolarizing steps in 10 mV increments from –90 to +20 mV. **(B)** Control (black) and response to 200 nM TTX (red) for nine calyces (P5–11) are shown in the IV plot of peak inward currents for steps between –80 and 0 mV. **(C)** Left panel: the residual current following 200 nM TTX is blocked in 1 μM TTX and the block reverses with washout in a P6 calyx. Currents in response to a voltage step from –130 to –30 mV. Right panel: 1 μM JZTX-III blocks a component of *I*_Na_ and the remaining current is abolished following application of 1 μM JZTX-III plus 200 nM TTX in a P7 calyx. Currents in response to a voltage step from –130 to –50 mV. **(D)** Summary for a group of 12 cells perfused with 200 nM TTX (six cells at P5–6, six at P8–11). A group of cells exposed to 1 μM TTX (*n* = 8, one cell at P6, seven cells at P7–10) *I*_Na_ was abolished in 1 μM JZTX-III and 200 nM TTX (*n* = 4, two cells at P6 and two cells at P7). Peak inward current was measured at –50 mV step. *I*_Na_ tended to be larger at younger ages as shown by distributions. In the presence of 200 nM TTX, *I*_Na_ decreased from –3.9 (1.8) to –0.25 (0.5) nA (*P* < 0.001, paired *t-test, n* = 12). Remaining *I*_Na_ ranged from 0.008 to 1.8 nA. In all cells exposed to 1 μM TTX, *I*_Na_ was completely abolished (*n* = 8, *P* = 0.001). *I*_Na_ was also completely abolished in a group of cells perfused with a combination of 1 μM JZTX-III and 200 nM TTX (*n* = 4, *P* = 0.022). External solution was L-15 in the TTX experiments, and 80 mM Na^+^ HEPES in the JZTX-III/200 nM TTX experiments; electrode solution Cs^+^. ^∗∗∗^*P* < 0.001, ^∗∗^*P* < 0.01, ^∗^*P* < 0.05.

In rodent vestibular end organs, hair cells and their afferent neurons are not mature at birth but continue to develop concomitant with vestibular function during the first few postnatal weeks (Curthoys, 1979; Rüsch et al., 1998; Lasker et al., 2008). We investigated the effect of the Na^+^ channel blocker TTX in calyces from postnatal pups, before physiological function of the vestibular system reaches maturation. Both isolated terminals and calyces in slices were studied; however, we did not distinguish between CZ and PZ calyces at this immature stage. As shown in Figure 6, perfusion of 200 nM TTX did not completely block the transient inward current in calyces at ages P5–11. Approximately 6% of peak *I*_Na_ following the voltage step to −50 mV remained in 200 nM TTX (Figure 6D; *n* = 12), suggesting the presence of Nav1.5, 1.8, and/or 1.9 channels during this stage of development. Application of 1 μM TTX completely abolished *I*_Na_ in immature calyces (Figure 6D) and the effect was reversible as shown for a P6 calyx with control current and currents in the presence of 200 nM TTX, 1 μM TTX, and recovery (Figure 6C). Application of 1 μM Jingzhaotoxin-III (JZTX-III), a blocker of Nav1.5 channels (Xiao et al., 2004), reduced *I*_Na_ by 16.3 (14) % (*n* = 4). The remaining current was abolished in a combination of JZTX-III and 200 nM TTX (Figure 6C, right, and Figure 6D, right). Since Nav1.8- and Nav1.9-mediated currents are resistant to block by 1 μM TTX, these observations strongly suggest that Nav1.5-mediated currents are present in immature calyces. As demonstrated in Figure 5, such currents were not present in calyces at older ages suggesting a transient postnatal expression of Nav1.5 channels. In agreement with our observations, staining for Nav1.5 channels was reported in calyces of the rat utricular epithelium at early postnatal ages (Wooltorton et al., 2007) and electrophysiological expression of Nav1.5-like *I*_Na_ was reported to be maximal during the first few postnatal days in isolated vestibular ganglion cells (Liu et al., 2016).

### TTX-Insensitive Currents in Early Postnatal Hair Cells

**FIGURE 7:**
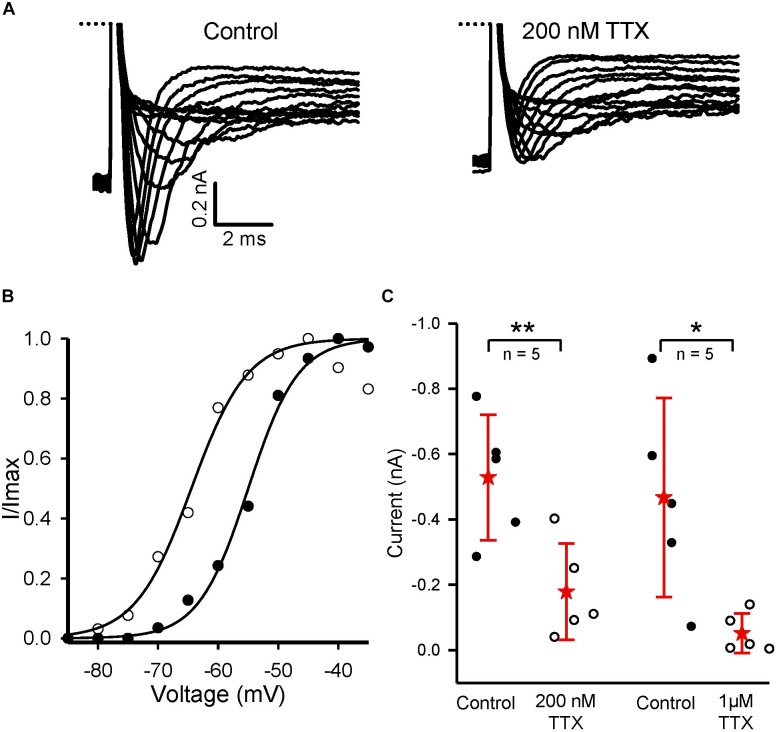
TTX-insensitive current is present in crista hair cells at early postnatal ages. **(A)** Control *I*_Na_ and currents in the presence of 200 nM TTX in a dissociated P5 hair cell in response to a series of voltage steps in 5 mV increments from –104 to –34 mV. **(B)** The activation curve for *I*_Na_ shifts to the left in 200 nm TTX (same cell as A, filled circles control, unfilled circles 200 nM TTX) indicating a more negative activation range for the TTX-insensitive current. **(C)** Mean reduction of inward current in 200 nM TTX for five pup hair cells (P5–9). Current decreased from a mean value of –0.53 (0.19) nA to –0.18 (0.15) nA, *n* = 5, *P* = 0.002, paired *t-test*. In 1 μM TTX, current decreased from –0.47 nA (0.3) nA to –0.052 (0.06) nA in five hair cells, all P5 (*P* = 0.038, paired *t-test*). ^∗∗^*P* < 0.01, ^∗^*P* < 0.05. Peak inward current was measured at the –50 mV step in eight cells, and at –45 or –30 mV in the remaining hair cells. External solution was L-15; electrode solution was Cs^+^-based.

We also investigated TTX-insensitive *I*_Na_ in early postnatal hair cells isolated from cristae. Immature vestibular hair cells express Na^+^ currents and have the capacity to fire action potentials during development. However, in birds and mammals, hair cells lose their Na^+^ currents and their firing ability with maturation (Sokolowski et al., 1993; Rüsch et al., 1998; Masetto et al., 2003; Géléoc et al., 2004; Wooltorton et al., 2007). Both TTX-sensitive and -insensitive Na^+^ currents were reported in hair cells of the rat utricle during the first three postnatal weeks and the TTX-insensitive current had biophysical characteristics consistent with Nav1.5 subunits (Wooltorton et al., 2007). In gerbil crista, the incidence of *I*_Na_ expression in hair cells was greatest during the first postnatal week (Li et al., 2010). We explored the sensitivity to TTX in immature hair cells and found that around one third of total I_Na_ remained in 200 nM TTX (Figure 7). In five hair cells (P5–9), 200 nM TTX blocked a component of *I*_Na_ (Figure 7C). In the presence of 200 nM TTX, the activation curve for *I*_Na_ shifted from −55 to −64.5 mV (Figure 7B), consistent with a TTX-insensitive current that activates at more negative membrane potentials. Immature crista hair cells are therefore similar to early postnatal utricle hair cells in their expression of TTX-sensitive and TTX-insensitive components (Wooltorton et al., 2007). In a group of five hair cells (all P5) exposed to 1 μM TTX, 88 (12) % of *I*_Na_ was blocked (Figure 7C). It is not yet known whether the current remaining in 1 μM TTX includes a TTX-resistant component mediated by Nav1.8 or 1.9 channels.

We conclude that a small TTX-insensitive *I*_Na_ is present in calyx terminals during the first and second postnatal weeks, but that this component is no longer present by the fourth postnatal week. Immature crista hair cells also show a TTX-insensitive *I*_Na_, similar to that seen in calyces and Nav1.5 subunits likely mediate the TTX-insensitive *I*_Na_ in both cell types.

### Nav1.6-Mediated Currents in Dimorphic Afferents

**FIGURE 8:**
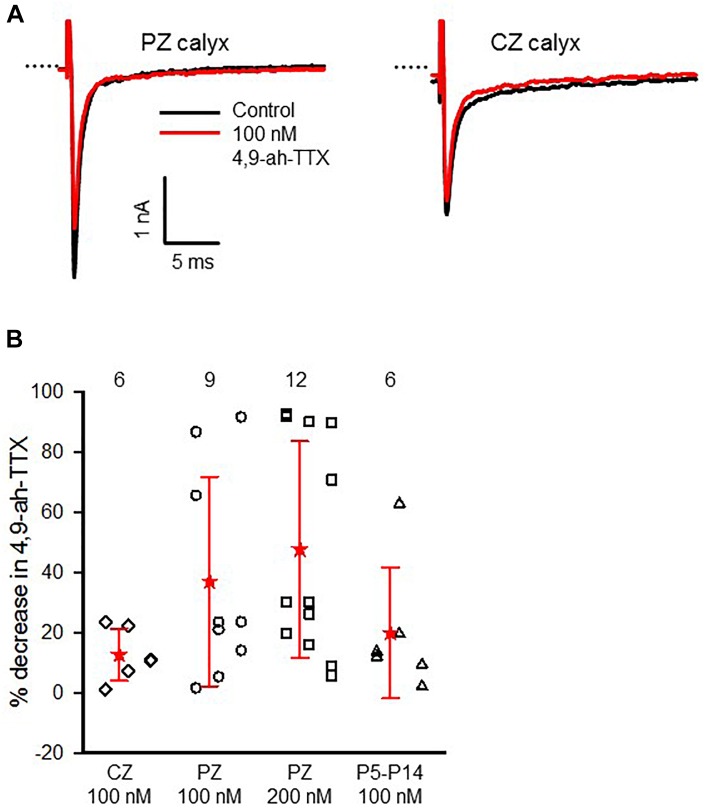
Effect of 4,9-an-TTX on *I*_Na_ is greatest in mature PZ calyces. **(A)** Reduction of *I*_Na_ in response to perfusion of 100 nM 4,9-ah-TTX in a representative PZ calyx (P31) and CZ calyx-only fiber (P27) in crista slice in response to a voltage step from –130 to –50 mV. External solution was 0 K^+^ HEPES. **(B)** Mean response to 100 nM 4,9-ah-TTX in mature CZ (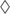; P20–27), PZ (o; P22–31) and immature calyces (Δ; P5–14) and response in mature PZ calyces (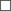; P22–29) to 200 nM 4,9-ah-TTX are shown. Numbers above plots indicate number of cells for each group. Electrode solution was Cs^+^-based and external solution was L-15 or 0 K^+^ HEPES with either 80 mM Na^+^ or 150 mM Na^+^.

**FIGURE 9:**
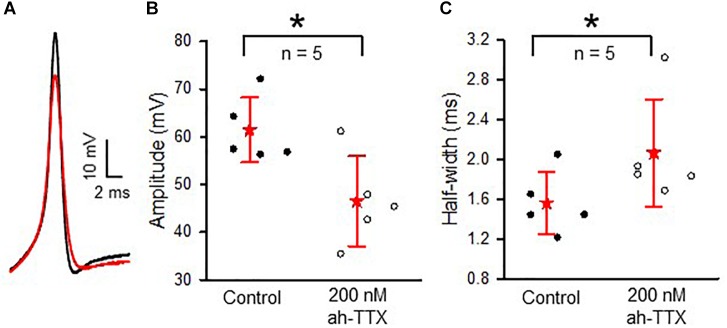
Contribution of Nav1.6-mediated current to action potential shaping in PZ calyces. **(A)** Averaged action potentials (*n* = 12 for each trace) in a PZ calyx (P21) in current clamp. Membrane was hyperpolarized briefly (–50 pA, 35 ms) to elicit action potentials in control conditions (black trace) and in the presence of 200 nM 4,9-ah-TTX (red trace). **(B)** In a total of five cells (ages P20-P25), application of 200 nM 4,9-ah-TTX decreased the mean action potential amplitude from 61.4 (6.8) mV to 46.5 (9.4) mV (*P* = 0.017) and **(C)** broadened action potential half-width from 1.56 (0.3) ms to 2.06 (0.5) ms (*P* = 0.014). ^∗^*P* < 0.05. For each calyx in **(B,C)**, an average of 6–30 action potentials was obtained. External solution was L-15, electrode solution was K^+^ based.

Immunoreactivity for Nav1.6 channels was reported in primary afferent fibers of the cochlea (Hossain et al., 2005; Fryatt et al., 2009; Kim and Rutherford, 2016) and vestibular system (Lysakowski et al., 2011), suggesting these subunits are important for conveying signals from the inner ear organs to the brain. We tested for the presence of Nav1.6 channels in vestibular afferents using 4,9-ah-TTX, an analog of TTX which is reported to selectively inhibit Nav1.6 channels in a range of cell types (Rosker et al., 2007; Teramoto et al., 2012; Hargus et al., 2013; Tsukamoto et al., 2017). We investigated the response of *I*_Na_ in calyces to 100 and 200 nM 4,9-ah-TTX. In pups (ages P5–7) 100 nM 4,9-ah-TTX reduced *I*_Na_ in only two out of seven calyces by 19 and 2%, respectively, whereas at ages P11–14, *I*_Na_ was reduced in all calyces tested, with a mean reduction of 24.4 (25.7) % (*n* = 4). In mature CZ terminals (P20–27), which were identified by fluorescent fills as calyx-only afferents, 100 nM 4,9-ah-TTX blocked only a small portion of the current in six out of nine cells and the mean reduction was 12.6 (8.7) % (*n* = 6). No reduction was seen in the remaining three cells. By contrast, 100 nM 4,9-ah-TTX reduced peak *I*_Na_ by 37.0 (34.8) % (*n* = 9) in PZ cells (Figure 8). In mature PZ calyces (P22–29), 200 nM 4,9-ah-TTX consistently reduced peak *I*_Na_ with a mean value of 47.7 (36) % (*n* = 12) and the block was reversible in 9 of the cells (not shown). In mature CZ calyx-only afferents (P21–P25), 200 nM 4,9-ah-TX did not block *I*_Na_ in two of three cells tested and blocked only 6.7% of *I*_Na_ in the third cell (data not shown). The greatest sensitivity to 4,9-ah-TTX was therefore seen in PZ calyces at older ages, suggesting that dimorphic terminals in mature crista have the greatest expression of Nav1.6 channels. We therefore investigated the role of these channels in action potential firing in PZ calyces. Action potentials were elicited in current clamp with brief hyperpolarizing steps and averaged as shown in Figure 9A. Evoked action potentials in PZ calyces were significantly reduced in height and increased in width in the presence of 200 nM 4,9-ah-TTX in 5/5 cells studied (Figures 9B,C).

We also investigated the effect of 4,9-ah-TTX in immature hair cells (ages P5–7, data not shown). Five out of six hair cells responded to 100 nM 4,9-ah-TTX with a decrease in *I*_Na_. The mean reduction was 18.2 (9.2) % (*n* = 5). These data are consistent with immunostaining for Nav1.6 that was described in hair cells of the early postnatal utricle (Wooltorton et al., 2007).

## Discussion

Primary vestibular afferent neurons are spontaneously active with action potential firing patterns varying from irregular to regular (Goldberg, 2000). Although firing is modulated by movements of the hair bundle which gate transduction channels, recent data suggest interactions between voltage-dependent channels within calyx terminals may also contribute to spontaneous firing. Specifically, firing in calyx terminals persists in the presence of blockers of mechanotransduction channels and α-amino-3-hydroxy-5-methyl-4-isoxazolepropionic acid (AMPA)-type glutamate receptors (Horwitz et al., 2014; Meredith and Rennie, 2015). We previously showed that transient inward currents in solitary calyces in gerbils P21 and older had rapid activation and inactivation properties, a half-inactivation of ∼ −83 mV and a range of peak current amplitudes (Rennie and Streeter, 2006). Here, we examined the identity and role of Na^+^ channel subunits underlying currents and action potential firing in calyx terminals from immature crista and in different regions of the mature crista. Our data revealed Na^+^ currents in calyx terminals with a range of kinetic properties and variable sensitivity to TTX and 4,9-ah-TTX. In mature epithelia, we discovered that *I*_Na_ in calyx terminals within the cista CZ showed slower inactivation kinetics, less negative half-inactivation potentials and smaller peak amplitudes compared to PZ terminals. We confirmed that large Na^+^ currents, including TTX-insensitive currents, are expressed in the first postnatal days and that the TTX-insensitive *I*_Na_ disappeared with development. At maturity, Nav1.6-mediated currents are present in calyces in both central and PZs of the crista, but make the largest contribution to *I*_Na_ in peripheral dimorphic fibers.

### TTX and 4,9-ah-TTX-Sensitive Sodium Currents

In mature calyces (after the third postnatal week), transient inward currents were abolished by up to 300 nM TTX in calyces in both zones of the crista, and we found no evidence for a TTX-insensitive component of *I*_Na_ at P21–29. We further investigated subunits contributing to the TTX-sensitive current and found that 4,9-ah-TTX, a selective inhibitor of Nav1.6 channels (Rosker et al., 2007), blocked a component of *I*_Na_ in both PZ and CZ calyces. In a previous report, Nav1.6 immunoreactivity was prevalent at the heminode and nodes of dimorphic, but not calyx-only, afferents (Lysakowski et al., 2011). We therefore tested the hypothesis that Nav1.6 channels make a larger contribution to the TTX-sensitive component of *I*_Na_ in PZ calyces and found that 200 nM 4,9-ah-TTX resulted in a ∼50% reduction of peak *I*_Na_ in PZ calyces but did not substantially reduce *I*_Na_ in identified CZ calyx-only terminals. At 100 nM, 4,9-ah-TTX blocked ∼37% of the current in PZ cells but only blocked a small portion (∼13%) of *I*_Na_ in a subset of identified calyx-only CZ cells. This is consistent with the strong Nav1.6 immunostaining at the heminode of central and peripheral dimorphic afferents in adult rat vestibular epithelia (Lysakowski et al., 2011). In maturing calyces (P11–P14), we found that 100 nM 4,9-ah-TTX reduced current in 4 cells by ∼25%, whereas at younger ages (P5–10), 4,9-ah-TTX produced a reduction in current in only 2/7 cells suggesting Nav1.6 is not prevalent at early postnatal days, but may be upregulated during development. This is similar to a recent report from mouse cochlea, where resurgent Na^+^ currents appeared in cultured spiral ganglion neurons at the end of the first postnatal week, became more prevalent around hearing onset (P12–14) and were sensitive to block by the Nav1.6 channel toxin 4,9-ah-TTX (Browne et al., 2017). Nav1.6-like immunoreactivity has also been described at the heminode of afferent fibers in the cochlea (Hossain et al., 2005; Kim and Rutherford, 2016) and 100 nM 4,9-ah-TTX blocked ∼70% of peak *I*_Na_ in cultured spiral ganglion neurons (Browne et al., 2017), suggesting that Nav1.6-mediated currents play a key role in action potential firing and conveying sound signals to the central auditory system.

In an initial report, 4,9-ah-TTX was reported to have much greater selectivity for Nav1.6 subunits compared to Nav1.2–1.8 subunits and to inhibit Nav1.6 channels with an IC50 of ∼8 nM when Na^+^ channel isoforms were expressed in *Xenopus* oocytes (Rosker et al., 2007). Subsequent studies with mammalian cells revealed IC50 values for expressed Nav1.6 channels between 100 and 294 nM (Hargus et al., 2013; Tsukamoto et al., 2017). Co-expression of Nav1.6 with β4 subunits resulted in resurgent Na^+^ currents (Grieco et al., 2005) and co-expression of β1 and β2 subunits in persistent Na^+^ currents (Smith et al., 1998). Beta subunits are single transmembrane cell adhesion molecules that modulate voltage dependence of activation and inactivation of *I*_Na_, inactivation kinetics, and channel density at plasma membrane (Isom, 2001). They also have non-conducting roles influencing cell adhesion, neurite outgrowth, and pathfinding processes (Kruger and Isom, 2016). The presence of β1-4 subunits has been reported in the vestibular ganglion (Liu et al., 2016) but their role in modulating Na^+^ conductances in calyx terminals remains to be determined.

In central neurons, Nav1.6 is highly expressed at the axon initial segment, where action potentials are generated (Hu et al., 2009). In globus pallidus and cerebellum, Nav1.6 channels have been implicated in autonomous pacemaker firing (Levin et al., 2006; Mercer et al., 2007). Given their prevalence in PZ afferents, the kinetics of Nav1.6 channels are likely to have a major influence on regulating excitability in these cells and using 4,9-ah-TTX we investigated the role of Nav1.6 channels in firing in PZ dimorphic afferents in current clamp. We found that action potential height decreased and width increased significantly in response to 4,9-ah-TTX. Faster inactivation of *I*_Na_ in dimorphic afferents expressing increased levels of Nav1.6 could promote rapid and tonic firing in these terminals. The *V*_1/2_ for inactivation was more negative in PZ afferents compared to CZ afferents and may reflect greater expression of Nav1.6 channels in dimorphs. Nav1.6 channels activate at relatively negative membrane potentials (O’Brien and Meisler, 2013), and their expression in PZ afferents may increase excitability by allowing more *I*_Na_ to be available at the resting potential. In Purkinje neurons, Nav1.6 channels play a crucial role in promoting repetitive and spontaneous firing (Raman et al., 1997). The greater prevalence of Nav1.6 channels in PZ terminals is consistent with the higher resting firing rates and regular firing patterns in these afferents (Goldberg, 2000; Eatock and Songer, 2011). In mice, regular afferents have an average resting firing rate of >60 spikes s^−1^ whereas irregular afferents fire at ∼40 spikes s^−1^ (Yang and Hullar, 2007). Our results in current clamp are reminiscent of the effects of 4,9-ah-TTX in medial entorhinal cortex layer II neurons. Following status epilepticus induction, Nav1.6 channels were upregulated in these cells and 4,9-ah-TTX reduced firing rates, decreased action potential amplitude, and increased the threshold and width of action potentials (Hargus et al., 2013). In a similar fashion, Nav1.6 channels may contribute to increased excitability of PZ dimorphs compared to CZ calyx only afferents.

The TTX-sensitive current component in vestibular calyx terminals was greater than current blocked by 4,9-ah-TTX, strongly suggesting that other TTX-sensitive subunits make contributions to *I*_Na_ in both calyx-only and dimorphic afferents. The identity of the remaining TTX-sensitive current in 100 nM 4,9-ah-TTX is unknown, but it may be carried by Nav1.1–1.3 and/or Nav1.7 subunits, which were detected in P21 vestibular ganglion neurons (Liu et al., 2016).

### TTX-Insensitive Sodium Current

Nav1.5 channels are moderately resistant to block by TTX, with IC50 values in the μM range (Goldin, 2001). Currents mediated by Nav1.8 and 1.9 channels are highly resistant to TTX block and remain even in micromolar concentrations of TTX. We previously reported that TTX (at 100 or 500 nM) blocked the vast majority of transient inward current in calyx terminals isolated along with their type I hair cells from the vestibular organs of gerbils (Rennie and Streeter, 2006; Meredith et al., 2011). Here, we confirmed that *I*_Na_ in calyces embedded in slices of mature crista is mediated by Na^+^ channels that are highly sensitive to TTX. However, at earlier stages of development, we found that a small current remained in the presence of 200 nM TTX in immature terminals. The TTX-insensitive *I*_Na_ represented about 6% of the total inward current and was abolished in 1 μM TTX, or by a combination of 1 μM JZTX-III plus 200 nM TTX, strongly suggesting it was mediated by Nav1.5 channels. Na^+^ currents sensitive to block by 1 μM TTX were also reported in cell bodies of the mouse vestibular ganglion at early postnatal stages P0–12 (Chabbert et al., 1997; Risner and Holt, 2006). However, a more recent study revealed the presence of additional Na^+^ current components that were not blocked by 300 nM and in some cases 5 μM TTX in rat vestibular ganglion neurons at P1–8 (Liu et al., 2016). RT-PCR probing provided evidence for the Nav channel subunits Nav1.1–1.9 in rat vestibular ganglia (Liu et al., 2016). Although these studies showed biochemical evidence for Na^+^ channels in cell bodies of the vestibular nerve, the types and regional distribution of Na^+^ channels within afferent terminals and axons, where spikes are initiated and subsequently propagated, was not evaluated. Our results support expression of Nav1.5-mediated currents, but not TTX-resistant currents (Nav1.8 and Nav1.9) within afferent terminals at early postnatal days. Immunoreactivity for Nav1.5 was shown on the inner face of calyces at older ages in rats (Lysakowski et al., 2011), but we found that the electrophysiological expression of TTX-insensitive current disappears with postnatal development and found no evidence for this current during the fourth postnatal week. It is unclear precisely when the TTX-insensitive component disappears since we did not systematically study cells in the third postnatal week.

Interestingly, there is no evidence for TTX-insensitive currents in early postnatal spiral ganglion neurons, since 100 nM TTX was reported to abolish *I*_Na_ in afferent neurons isolated from mouse and rat cochlea at P8–9 (Valdes-Baizabal et al., 2015; Browne et al., 2017). Nav1.5-like currents have now been reported during the early postnatal period in hair cells, calyx terminals and cell bodies of the vestibular ganglion suggesting that they play an important role for the development of the vestibular periphery. Nav1.5 channels are also reported in the olfactory epithelia, where they are expressed in sensory neurons at the apical knob, but not at other sites along the axon (Frenz et al., 2014). Spontaneous firing was inhibited by blocking Nav1.5 channels in olfactory neurons (Frenz et al., 2014). The role of Nav1.5-mediated currents in firing in developing calyces remains to be addressed.

### Na^+^ Currents in Vestibular Hair Cells

Voltage-gated Na^+^ currents are prevalent in rodent vestibular hair cells during embryonic and postnatal development (Lennan et al., 1999; Chabbert et al., 2003; Géléoc et al., 2004; Wooltorton et al., 2007; Li et al., 2010). During this period, vestibular hair cells emerge into phenotypically distinct type I and type II populations and reach electrophysiological maturity during the first postnatal month (Rüsch et al., 1998). Mature mammalian hair cells express diverse K^+^ currents but no longer display Na^+^ currents (Meredith and Rennie, 2016). Na^+^ currents are maximal around birth, but absent in hair cells by postnatal week 4 in utricle (Géléoc et al., 2004; Wooltorton et al., 2007) and by postnatal week 6 in gerbil crista (Li et al., 2010). TTX-sensitive and TTX-insensitive Na^+^ current components have been identified in developing hair cells of rat utricle. The TTX-insensitive component was present in all type I hair cells and approximately half of type II hair cells in the third postnatal week and appeared to be mediated by Nav1.5 channels (Wooltorton et al., 2007). We found evidence for TTX-sensitive and -insensitive currents in crista hair cells, with approximately one-third of *I*_Na_ remaining in 200 nM TTX at P5–9. The TTX-insensitive current was largely blocked by 1 μM TTX strongly suggesting it was mediated by Nav1.5 channels. The activation *V*_1/2_ shifted to more negative potentials in 200 nM TTX, which is also consistent with the more negative activation of Nav1.5 currents. Interestingly, Nav1.5-like immunoreactivity was observed in both utricular hair cells and calyces up to P21, suggesting these channels may be present on both sides of the synaptic cleft (Wooltorton et al., 2007). The Na^+^ channel subunits responsible for the TTX-sensitive current in developing hair cells were not identified previously. Our results showing partial block of *I*_Na_ in hair cells by 100 nM 4,9-ah-TTX suggest Nav1.6 subunits also contribute to the TTX-sensitive current in developing hair cells.

### Na^+^ Currents in Vestibular Ganglion Neurons

Within the ear, action potentials are thought to be initiated in the spike initiation zone of nerve terminals close to the sensory hair cells (Hossain et al., 2005) and subsequently propagate along axons and cell bodies to the CNS. Several conductances have been characterized in the afferent cell bodies of the vestibular ganglion including Na^+^ and K^+^ conductances. Na^+^ currents and firing have been studied in the cell bodies of early postnatal vestibular ganglion neurons before they become extensively myelinated (Chabbert et al., 1997; Risner and Holt, 2006; Cervantes et al., 2013; Liu et al., 2016). In a detailed characterization of Na^+^ currents in P3–8 dissociated ganglion neurons, Liu et al. (2016) demonstrated TTX-sensitive, TTX-insensitive, and TTX-resistant current components. Nav1.5-mediated current persisted in 300 nM TTX in some neurons, but was abolished in 5 μM TTX. In another group of ganglion cells, a component of *I*_Na_ persisted in 5 μM TTX and was hypothesized to be mediated by Nav1.8. Our data support a Nav1.5-mediated current in early postnatal calyces, but *I*_Na_ was completely blocked by 1 μM TTX and we found no evidence for a TTX-resistant current in immature or mature calyces.

The findings reported here have advanced our understanding of the identity and role of specific groups of Na^+^ channels in driving afferent excitability. Drugs targeting Nav channels are commonly used as local anesthetics, analgesics, anti-convulsants, and anti-arrhythmics. In nociception pathways, Na^+^ channel subunits can be selectively targeted by drugs with the goal of alleviating the sensation of pain (Emery et al., 2016; Luiz and Wood, 2016). In a similar fashion, Na^+^ channel modulators could be used to alleviate debilitating vestibular symptoms by targeting specific types of Na^+^ channels in vestibular afferents. Knowledge of underlying subunits and their roles in firing also has implications for vestibular implants, where electrical stimulation of the vestibular nerve evokes action potentials and attempts to mimic natural stimuli (Lewis, 2016).

## Author Contributions

FM and KR performed the research, designed the experiments, and analyzed the data. FM prepared the figures. KR wrote the first draft of the paper.

## Conflict of Interest Statement

The authors declare that the research was conducted in the absence of any commercial or financial relationships that could be construed as a potential conflict of interest.
